# An empirical study of schema-associated mnemonic method for classical Chinese poetry in primary and secondary education based on cognitive schema migration theory

**DOI:** 10.1038/s41598-023-47826-x

**Published:** 2023-11-25

**Authors:** Dawei Liu, Ping He, Huifen Yan

**Affiliations:** 1 College of Teacher Education, Hubei University of Education, Wuhan, 430205 China; 2 College of Foreign Languages and Literature, Wuhan University, Wuhan, 430072 China; 3 Institute of Educational Sciences, Wuhan University, Wuhan, 430072 China

**Keywords:** Cognitive ageing, Cognitive neuroscience

## Abstract

Classical Chinese poetry, as an indispensable part of China′s national culture, provides valuable resources for improving literary literacy and fostering patriotism among primary and secondary school students. This study, based on cognitive schema migration theory, investigated the effect of a "schema-associated mnemonic method" on the memory efficiency and retention of classical Chinese poetry memorization among the students. Through a controlled experiment, the results revealed that the experimental group (n = 63) outperformed the control one in memorizing classical poetry with higher memory efficiency and retention, which indicates the sustainable application prospects of the method in primary and secondary education. The findings of this research can provide beneficial insights for the effective integration of classical Chinese poetry teaching and educational technologies.

Classical Chinese poetry is greatly cherished in Chinese culture. Memorizing classical poems is helpful for students to enhance their aesthetic tastes and cultural literacy, foster patriotism, and promote national culture. By virtue of poetry memory, students′ cultural perception can be cultivated and enhanced, and their cultural consciousness can be activated, which can further render the effectiveness of cultural education more significant^1^. It is explicitly required in the *Chinese Curriculum Standards of Compulsory Education* (2011-Ed)^2^, hereinafter referred to as the "Standards") that primary and secondary school students should memorize a total of 240 excellent poems and prose passages. Through conscious accumulation, perception, and application of these works, students are enabled to improve their appreciation tastes and aesthetic views. Moreover, the Standards also points out that the examination focus of classical-poetry part lies in students′ memory accumulation. More attention should thus be paid to the memory of classical poetry in both the teaching and learning process of Chinese.

As Francis Bacon once remarked, "All knowledge is but remembrance." Since memory is the foundation of learning, more importance should be attached to it in Chinese learning^3,4^. Good memory not only enriches the accumulation of knowledge but also serves as an effective method to improve reading, writing, listening, and speaking abilities^5^. It also contributes to intellectual development and nurtures the spirit of innovation^6^, enabling one to "be eloquent with a myriad of words and phrases in the mind."

Regarding the memory of classical Chinese poetry, different scholars have proposed various memory methods, including Situational Induction^7,8^, Combination of Poetry and Painting^9,10^, Reproduction of Poetic Field^11^, and Association of Image^12,13^. However, the majority of the published articles on the study available currently merely illustrate their standpoints combining with individual teaching experience, with little empirical research on the practical effects of specific mnemonic methods for classical Chinese poetry. In light of the author′s teaching and training experience, this manuscript proposed a method for reciting Chinese poem, namely, Schema-Associated Mnemonic Method for Classical Chinese Poetry. This method is put forward according to memory techniques proposed by previous scholars^14–16^ who emphasized that association and imagination are the two most important memory strategies when memorize new things. It is also accords with the widely acknowledge view that knowledge is organized in long-term memory in non-linear forms^15^, as well as the constructivist view that existing knowledge has great impact on future learning through integrating prior knowledge with new knowledge structures to form more sophisticated schema^17^. This attempts to present a more effective way of memorizing classical poetry to improve primary and secondary school students′ memory efficiency and retention of classical poetry. Meanwhile, it aims to increase students′ interest of and participation in learning Chinese classical poetry and provide new insights and practical value in the field of poetry education.

In light of this fact, based on Ausubel′s cognitive schema migration theory^17^, this study adopts an experimental approach to examine the effects of the schema-associated mnemonic method for classical Chinese poetry on memory efficiency and retention. It intends to fill the gap of lack of empirical research on the memory retention and efficiency of classical poetry by using specific mnemonic method. By deeply understanding the practical effects of the schema-associated mnemonic method in the teaching and learning of Chinese poem, we aim to provide more powerful poetry educational technology support and guidance.

The following research questions will be addressed: (1) How is the memory efficiency of the schema-associated mnemonic method for classical Chinese poetry? (2) How does the method affect the retention of classical poetry memory?

Through in-depth research on these issues, we can provide empirical evidence and effective application strategies for the teaching and learning of Chinese classical poetry. This can further improve students′ memory efficiency and enhance their interest in learning poem, which could offer vitality and vigor to the domain of poetry education. Additionally, this study is hoped to provide new insights into the integration of cognitive theory and educational practice, and shed new lights on future educational research and practice. Meanwhile, we will explore the use of this method in other education arena to promote the effectiveness and sustainable application of educational technology.

## Cognitive schema migration theory and schema-associated mnemonic method

### Cognitive schema migration theory

According to Ausubel^18^, the cognitive structure of learners refers to the knowledge structure in their minds. In a broader sense, cognitive structure refers to the content and organization of all the ideas possessed by learners, while in a narrower sense, it refers to those within a specific domain or discipline^17^. Each learner has his unique cognitive structure of which the characteristics in terms of content and organization are referred to as cognitive structure variables, including generalizer and particularizer^17,19^. Ausubel^20^ argues that the learning of new materials must be based on the learner′s existing knowledge. The learning process involves establishing nonarbitrary and substantive (or nonverbatim) connections (i.e., assimilation) between new materials and the appropriate existing relevant ideas in the learner′s cognitive structure^19^. Therefore, reinforcing the existing cognitive structure can facilitate the learning of new materials, and the great extent to which new materials are connected to the existing cognitive structure distinguishes meaningful learning from rote learning^20^. Furthermore, the acquisition and retention of knowledge result from active and comprehensive interacting processes between new materials and the relevant content within the learner′s cognitive structure. Meaningful learning, compared to rote learning, is of higher efficiency and can enhance memory retention^18^.

Transfer refers to "the influence of one learning on another"^21^. It occurs when the existing cognitive structure influences new cognitive functions^17^. Thus, the transfer can be classified into forward transfer (the influence of the previous cognitive structure on current learning) and backward transfer (the influence of new concepts and information on the existing cognitive structure). Depending on its effect, the transfer can be positive (facilitating) or negative (interfering)^17^. It is achieved through the operation of cognitive structures and influenced by three variables of cognitive structure: availability, discriminability, and stability^17–20^. The variables are positively correlated with transfer, manifested in that the higher availability, discriminability, and stability, lead to higher generality, clarity, and stronger consolidation of the existing concepts, resulting in better transfer effects eventually. Therefore, in learning, "advance organizers" can be designed to modify cognitive structure variables, bridge the gap between the learner′s existing cognitive structure and new materials, thereby promoting learning efficiency and memory retention in an ideational-scaffolding manner^19,22^.

In summary, the learning process is a transfer process, and meaningful learning involves connecting new materials with existing knowledge content, and integrating new knowledge into the learner′s existing cognitive structure. Thus, through optimizing the learner′s cognitive structure, learning efficiency and memory retention can be both improved.

In conclusion, learning process is a process of transfer. The most important change in the cognitive system during learning is the transfer of information from working memory to long-term memory through deep processing. In the procedure of information processing and cognitive processing, only by transforming working memory into long-term memory can learners′ knowledge schema be formed, so as to stabilize and retain the acquired knowledge for a long time. With the help of schema, learners can quickly establish connections between new knowledge and concepts and existing schema, forming new cognitive structures. This can in turn help learners better understand and remember new knowledge and promote the transfer and application of knowledge from among different fields.

### Schema-associated mnemonic method for classical Chinese poetry

Bartlett^23^ defined a schema as "an active organization of past reactions or experiences", and Rumelhart^24^ further proposed that units, chunks, and systems composing the knowledge stored in the human brain are exactly schemas. Thus, schemas are the existing knowledge structures in learners′ minds and can be considered as a type of cognitive structure^25^. Moreover, schemas are the forms in which knowledge exists in long-term memory. The automation of schemas and rules does not require additional resource consumption, thereby compensating for the limited capacity of working memory and reducing the cognitive load^25^. Consequently, strengthening the construction of background-knowledge schemas in teaching activities can help learners establish a sound cognitive structure and facilitate the learning of new knowledge^26,27^.

Association, specifically, involves transforming classical poetry into a vivid image through the existing cognitive structure. In the schema-associated mnemonic method, new materials are connected with familiar background-knowledge schemas, thereby reinforcing memory^28^. The rich colors and vivid images involved in schema-associated memory can capture learners′ attention, render them focused, and bring them a sense of belonging in the Chinese class^29^, thus aiding the improvement of memory efficiency. Furthermore, the schema-associated mnemonic method fully leverages learners′ autonomy and initiative^28^. By combining with individual cognitive styles, learners can create images that are exclusively suitable for their memory, thereby enhancing memory effects^29^.

In the case of classical Chinese poetry, generally, a poem often intertwines various factors such as the poet′s sentiments and background, and the social environment, making itself easier to be transformed into concrete images through cognitive schema associations. When memorizing the poems, learners can immerse themselves in the emotional context, increase the relevant cognitive load, and relate the new poetry to their existing life experiences and knowledge^30^. By linking the newly learned classical poetry with existing cognitive schemas, cognitive assimilation is achieved^31^, through which the migration of cognitive structures can be promoted^29^.

**Figure 1:**
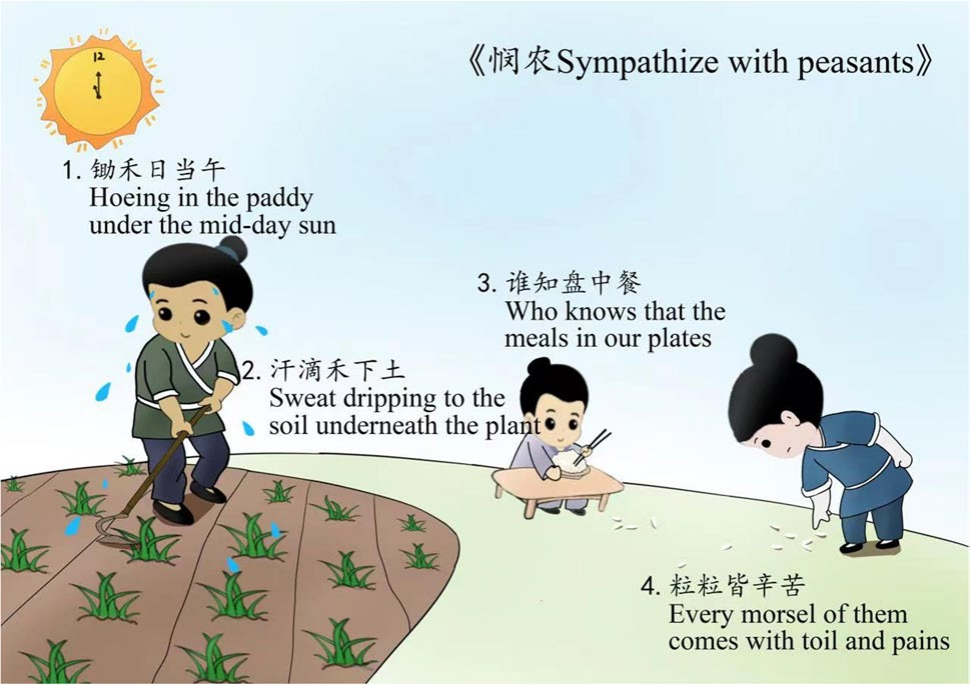
Schema-associated mnemonic method example.

Therefore, the schema-associated mnemonic method establishes a connection between new poems and existing background-knowledge schemas as well as cognitive structures, facilitating the positive transfer of cognitive structures and reinforcing relevant cognitive load. Additionally, the involvement of imagery in memorizing process helps reduce external cognitive load, capture learner′s interest, and enhance their subject status, eventually leading to the improvement of memory efficiency and retention. Figure 1 depicts an image created with the schema-associated mnemonic method. Even learners who have not previously been exposed to this specific poem can quickly memorize it by combining their preexisting cognitive schemas with the image. In the image, the sun with a clock symbolizes "midday" and the man with a hoe in the farmland represents "hoeing in the paddy under the mid-day sun"; the sweat from the man into the field of grain seedlings suggests "sweat dripping to the soil underneath the plant"; the child eating the meal at the table represents "who knows that the meal in our plates"; and the woman bending over and picking up rice grains marks "every morsel of them comes with toil and pains". Through the imaginative representation of the image combined with the poem title "Sympathize with peasants" as well as the existing knowledge about another familiar verse line, “Who knows that the meals in our plates? Every morsel of them comes with toils and pains”, learners can gain a rough idea of the main themes of this poem, namely the arduous lives of peasants at the time, sympathy for the peasants, and appealing for cherishing grains. Thus, the mnemonic method can help learners finish rapid memorization while incorporating emotions, consequently reinforcing cognitive structures and transfer, and enhancing memory retention.

## Research methodology

This study adopted a comparative experimental study, including the control group and experimental group. Students from experimental group employed the schema-associated mnemonic method during the test, while those from control group did not use this method in the test. Therefore, we could make a comparison of the memory efficiency and retention between the two groups. It could also provide evidence for the effectiveness and efficiency for this new method proposed by us.

### Research participants

Initially, it should be noted that the authors have gotten the oral consent of all the participants, all methods were carried out in accordance with relevant guidelines and regulations, and all the processes of this study have been approved by Brain Science and Learning Science Committee of Hubei Teachers Education Association.

This study selected normal students from five classes of a university located in Wuhan as the research participants, including three freshmen classes and two senior classes (characterized by multi-disciplinary). Given the fact that the freshmen had just finished the college entrance examination and had a certain amount of accumulation of classical Chinese poetry, they were set as the control group, while the senior students were set as the experimental group. Both groups had not received any classical Chinese poetry schema training before taking part in this experiment. The control group (n = 64) comprised 40 female students and 24 male students, with an average age of 19 years old; the experimental group (n = 63) was constituted of 55 female students and 8 male students, with an average age of 22 years old.

### Experimental procedure

#### Testing material

To ensure a better reliability and validity, we have made a pilot test to choose the appropriate poems for the current study. Also, the lexical complexity and sentence structure of the poems were also taken into consideration during the selecting process. Therefore, quite unpopular seven-character quatrains were selected as the testing material and these poems did not appear in students′ textbooks or common poetry anthology to reduce the interference of students′ existing knowledge on the memory process. Moreover, during the testing process, participants were required to skip those poems that they have known before (if any) to ensure that participants have not met all the selected poems before the test. In terms of the difficulty level of these poems, we chose those with challenges but not too much complexity based on the language proficiency and literature background of the participants. This can balance the difficulty level of poems and completion degree of memorization. In order to achieve this, we have conducted a pilot test to determine the appropriate difficulty level of the selected poems.

#### Testing procedure

The experiment lasted for eight school weeks. The control group did not receive any interventions, while the experimental group underwent schema-associated mnemonic method training once a week for the first six weeks with each session lasting for three hours and covering principles of the method, practice exercises, and the application of the method in teaching. In the seventh week, both the experimental and control groups went through the first round of tests. The testing materials consisted of 12 classical poems that the students had not previously memorized. For the control group, the students were provided with only the poems′ titles, authors, and original texts during the memorization process. In contrast, the experimental group students were given extra memory aids in the form of imagery (as shown in Fig. 1) along with the same materials. The memorization time was set to 10 min, during which the students were required to memorize the poems in sequential order. Only after having completely memorized one poem were they allowed to proceed to the next. Immediately after the memorization, the students were asked to write down the memorized poems, with a time limit of 20 min. The scoring was based on the quantity of correctly-written lines, with each line worth 2 points provided that the consecutive correct words equaled or exceeded half of the original line length (e.g., for a seven-character quatrains, obtaining four or more consecutive correct characters would earn 2 points). If the consecutive correct words were less than half of the original line length, 1 point was awarded. But complete errors or blank responses received 0 points. The scores were accumulated accordingly. After a seven-day interval, the second round of tests was conducted in the eighth week. The students were gathered and asked to directly write down the poems memorized in the seventh week within a time limit of 20 min, and the scoring criteria remained the same. Following the completion of the seventh-week testing, the memorization materials were immediately collected without informing the students about the upcoming second round of tests in the following week, thereby reducing the impact of students′ review on memory retention.

After the students finished the second round of tests, ten students from the experimental group were randomly selected for retrospective interviews, which were aimed at reviewing their memory processes and gathering insights into the application of the schema-associated mnemonic method for classical Chinese poetry.

### Data collection and analysis

After the completion of the two rounds of tests, the scores, number of complete poems and lines in the written responses of both the experimental and control groups were collected and analyzed. The first round of tests reflected the impact of the schema-associated mnemonic method on memory efficiency, while the second round revealed its effect on memory retention. The testing data were disposed of with Excel, and the scores and quantity of complete lines of both groups were analyzed with SPSS. After conducting the normality test, it was found that the data for the experimental group did not follow a normal distribution (*p* < 0.05). Therefore, the Mann–Whitney U test was employed. Additionally, to gain a comprehensive understanding of the learners′ memory performance, the mean, median, mode, and standard deviation of the scores and the number of complete lines were calculated.

For the interview data, content analysis was conducted to summarize how the students adopted the schema-associated mnemonic method, as well as their attitudes toward the method, which was also helpful in understanding the process of cognitive transfer among the learners. In view of a good group homogeneity of the participants and the writing from memory method as a classical test for memory studies, we hold the view that there is a good reliability and reliability. Therefore, we did not conduct further reliability and validity test.

### Ethical approval

This study have been approved by Brain Science and Learning Science Committee of Hubei Teachers Education Association.

### Informed consent

Informed consent was obtained from all study participants involved in the study.

## Results and discussion

### Memory efficiency of the schema-associated mnemonic method for classical Chinese poetry

As shown in Table 1, there was a significant difference in the efficiency of poetry memorization between the control and experimental groups (Z =  − 8.032, *p* < 0.05), indicating that the schema-associated mnemonic method can effectively enhance the efficiency of classical Chinese poetry memorization. The experimental group students were able to correctly remember a greater amount of poetry content within the given time frame. In terms of the mean scores, the control group had a mean score of 13.09, while that of the experimental group reached 30.29, twice over the control group′s mean score. The median reflects the overall level of the data. The control group had a median score of only 10.5, while the experimental group had a median score of 30, nearly three times that of the former The mode is the most frequently occurring value in the data. The control group had a mode of 8 while that of the experimental group was 32, four times as large as the control group′s mode. Overall, the experimental group obtained significantly higher scores than the control group, indicating that the schema-associated mnemonic method can help students remember a greater amount of poetry content within the given time frame, thus improving memory efficiency.

**Table1:** Effect of schema-associated mnemonic method on memory efficiency.

	Mean	Median	Mode	Standard deviation	Z value	*p* Value
Control group	13.09	10.5	8	8.85	− 8.032	0.000
Experimental group	30.29	30	32	9.22		

The assessment of poetry memory efficiency should not only take into account the amount of content but also emphasize the number of fully-memorized lines. As shown in Table 2, there was also a significant difference in the efficiency of fully memorizing lines of classical poems between the control and experimental groups (Z =  − 5.960, *p* < 0.05), revealing the facilitative effect of the mnemonic method on the memory efficiency, because the experimental group had higher mean scores, medians, and modes compared to the control group. Additionally, the average number of fully-memorized lines by the experimental group was 11.32, which was approximately twice that of the control group (5.92 lines).

**Table 2:** Efficiency of fully memorizing lines of the poems.

	Mean	Median	Mode	Standard deviation	Z value	*p* Value
Control group	5.92	5	4	4.12	− 5.960	0.000
Experimental group	11.32	11	7	5.33		

Furthermore, as for the efficiency of fully memorizing poems (as shown in Table 3), there were 38 participants in the control group who could remember 1 or 2 complete poems and only 3 participants who could remember 3 or more. The percentage of participants who could fully memorize poems was 59.38%. In contrast, the experimental group had 28 participants who could remember 1 or2 complete poems and 15 participants who could remember 3 or more. The percentage of participants who could fully memorize poems was 68.25%. Therefore, the schema-associated mnemonic method can also improve the efficiency of fully memorizing poems to some extent. For example, in the experimental group, 2 participants were even able to remember 6 complete poems within the 10-min time frame.

**Table 3:** Efficiency of fully memorizing poems.

	1–2 Pieces (percentage)	≥ 3 Pieces (percentage)	Total (percentage)
Control group	35 (54.69%)	3 (4.69%)	38 (59.38%)
Experimental group	28 (44.44%)	15 (23.81%)	43 (68.25%)

Based on the above analysis, it can be concluded that the schema-associated mnemonic method does enhance the efficiency of memorizing classical Chinese poems, allowing for the memorization of a greater amount of poem content within a given time frame, including both the number of fully-memorized lines and poems. This effect is attributed to the use of background-knowledge schemas guided by the method, which students in the experimental group have learned and mastered. Consequently, they have cognitive structures be formed in their minds, which can facilitate the connections of new materials with existing knowledge^20^, thereby reinforcing cognitive structures in turn^19^ and promoting active interaction between newly-acquired knowledge and pre-existing cognitive structures, ultimately aiding in memorization^18^. The interaction reduces the psychological burden and promotes the automation of schemas, compensating for the limited capacity of working memory and lessening cognitive load, and eventually enhancing memory efficiency^25^.

The schema-associated mnemonic method assists learners in constructing mental scenes through association, evoking their emotions and enabling them to memorize classical poems in an immersive manner, thus fostering emotional resonance with the poets^3,7,8^. In this way, learners can establish strong associations with their existing cognitive experiences and knowledge, which can facilitate the migration of cognitive structures and aid in memory. Furthermore, learners can incorporate their own real-life experiences^10,30^ though association and imagination^14–16^ and even scenes from relevant movies, to regulate the cognitive load and promote the construction and automation of cognitive schemas, thereby enhancing memory performance^32^. As evidenced by the retrospective account of the first interviewee regarding the memorization process, "*I would associate familiar characters with costume dramas to make the scenes more memorable with their strong relevance*."

Furthermore, students in the experimental group were enabled to have their memory efficiency be improved by means of existing memory images which are characterized by rich colors and vivid contents, and can help learners be immersed^29^ and facilitate memorization through the integration of poetry content and visual elements^9,13^. Additionally, image-based memory reduces the difficulty of to-be-memorized content, thus reducing the external cognitive load during memory retrieval^32^ and enhancing memory efficiency. Images also aid learners in quickly retrieving information during recall, leading to more robust memory^12^.

From the interview content, it can be observed that nine interviewees employed the techniques of “constructing scenes, comparing images, and matching corresponding text”, indicating the practical application of the schema-associated mnemonic method. Additionally, one interviewee adopted a technique of “image with rote memorization”. During the recall process, the ten interviewees all followed the procedures of “recalling the images, reorganizing the sequence, and extracting the corresponding text”. For example, one of the interviewees stated, "*First, I thought of the images as well as their sequence, then I extracted the key points accordingly and wrote them down* (S8)." When asked about whether image-based memory took effects, all the interviewees responded affirmatively, by stating that image-based memory helps them save memorizing time and aids in the recall. As long as they can recall the images, they can generally recollect the content. These findings reveal that the schema-associated mnemonic method effectively helps learners improve memory efficiency by combining images with scenes, reducing memory time, and enhancing the accuracy of recalled content. Meanwhile, eight interviewees believed that there is a strong relevance between the efficiency of memorizing classical Chinese poems and the amount of pre-existing knowledge accumulation. And they found that employing the mnemonic method did work on helping them relate the poems to prior knowledge. For instance, one of the interviewees stated, "When memorizing the first two poems, *A Soldier′s Complaint* and *To the Returning Wild Geese*, I immediately thought of various frontier fortress poems and lines like ′the Yellow River soars into the white clouds′ and ′For vernal wind never blows over Yumen Pass′ (S3)." It shows that the mnemonic method also aids learners in associating with pre-existing cognitive background schemas, and promotes the positive transfer of cognitive structures.

It should be noted that cognitive schema is characterized by variable, structural, and active^33^, and cognitive structures often exhibit individual differences^19^. Therefore, memory processes may vary with each learner, resulting in different memory outcomes, as evidenced by the experimental group′s scores ranging from 12 to 48 points. This also points to the learners′ autonomy^28^, as the schema-associated mnemonic method requires learners to exert their initiative and utilize techniques of emotions, keywords, images, etc. to establish connections between new materials and pre-existing cognitive structures so as to reinforce memory effect.

### Memory retention of the schema-associated mnemonic method for classical Chinese poetry

According to Table 4, there is a significant difference in the retention of memorized poems between the control group and the experimental group (Z =  − 7.865, *p* < 0.05), which indicates that the schema-associated mnemonic method can effectively enhance the retention of memorized poems. One week later, students in the experimental group were able to recall a greater amount of poem content correctly, with an average retention rate of 59.99%, which is remarkably higher than that of the control group (37.93%). The mean, median, and mode scores of the experimental group were all higher than those of the control group. The overall level of scores and the most common score in the experimental group were respectively 17 and 16 points, while in the control group, the numbers are 3.5 and 0, demonstrating that the experimental group had better memory retention and was able to retain more memory content over the course of a week.

**Table 4:** Effect of schema-associated mnemonic method on memory retention.

	Mean	Median	Mode	Standard deviation	Average retention rate (%)	Z value	*p* Value
Control group	5.34	3.5	0	5.30	37.93	− 7.865	0.000
Experimental group	17.97	17	16	8.35	59.99		

Furthermore, as displayed in Table 5, there is also a significant difference in the retention of fully-memorized lines of classical Chinese poems between the control group and the experimental group (Z =  − 4.129, *p* < 0.05). This reveals the facilitating effect of the schema-associated mnemonic method in improving the retention of fully-memorized lines of the poems. Although both the control group and the experimental group had the highest number of participants who were not able to write any single complete line (i.e. both modes were 0), the medians showed a persuasive difference with that of the control group being 1 (line), while the median of the experimental group reaching 5 (lines). A significant difference also emerged in the average number of fully-memorized lines between the two groups, with the control group′s mean value being less than half of that of the experimental group (2.25 vs 4.90). Therefore, in terms of the retention of fully-memorized lines, the experimental group is markedly higher than the control group, with the average retention rates being 44.85% and 32.05%, respectively, which demonstrates that the schema-associated mnemonic method can enhance memory retention and promote the transfer of cognitive structures.

**Table 5:** Retention rate of fully-memorized lines.

	Mean	Median	Mode	Standard deviation	Average retention rates (%)	Z value	*p* Value
Control group	2.25	1	0	2.58	32.05	− 4.129	0.000
Experimental group	4.90	5	0	3.67	44.85		

It is worth noting that although there is a significant difference between the control group and the experimental group in terms of the retention of memorized content and fully-memorized lines, no significant difference appears in the retention of fully-memorized poems. There were 12 participants in the control group who were able to write 1–2 complete poems, while in the experimental group, the number was 20, with the rest students unable to completely write down any single poem.

As stated above, the schema-associated mnemonic method can markedly enhance the retention of memorized content and fully-memorized lines of poems, and partly improve the retention of fully-memorized poems. This reveals the advantage of the method as a meaningful-learning oriented one over rote learning: meaningful learning establishes nonarbitrary and substantial connections between new content and the existing conceptual framework in the learner′s cognitive structure, achieving cognitive assimilation and the migration of cognitive structure^19^, which eventually aids in memory retention. In contrast, rote learning separates from the cognitive structure and is susceptible to interference from similar content, which is not conducive to memory retention^20^. Therefore, the experimental group exhibits higher memory retention.

Moreover, the experimental group students have received training about the mnemonic method, which serves as an "advance organizer" and bridges the gap between learners′ existing cognitive structures and new materials, thus facilitating memory retention with an "ideational scaffolding"^19,22^. Additionally, the "advance organizer" enables learners to enhance the availability and discriminability of their cognitive structures, thereby facilitating the positive transfer^17–20^ and reinforcing memory retention. Furthermore, the mnemonic images in memory formation, through their association with existing cognitive schemata, promote cognitive assimilation^28^ and cognitive transfer^29^, thereby aiding in memory retention. Responses from the interviewees provide endorsement for this finding, as the 10 interviewees all acknowledged that visual imagery contributes to their memory retention, and stated that the recall of the images directly led to the recollection of poems content. For example, one of the interviewees mentioned, “*As long as familiar scene images are constructed in my mind, memorizing things will be simpler, and with better retention*” (S1).

However, it should be highlighted that the experimental group did not demonstrate a significant advantage in terms of the quantity of fully-memorized poems. This can be attributed to that, despite the integration of prior knowledge with new knowledge, leading to the formation of more complex cognitive schemata^17^, the learners did not engage in any review or consolidation within the one-week period, resulting in insufficient stability of their cognitive structures and partial loss of the memorized content. This is evident in the response of the fourth interviewee who stated, "*If we had made a review, it would have been beneficial for memory retention. But we didn′t review the training content after class, so now, I don′t remember anything. However, I believe that if I could take a look at the images again, I will be able to quickly recall everything* (S4).”

In summary, the experimental group outperformed the control group in both test sessions, indicating that the schema-associated mnemonic method effectively enhances the efficiency and retention of classical poem memorization. By adopting techniques of association, imagery, emotions, colors, key points, combining painting and poetry, etc., the mnemonic method facilitates the connection between new materials and learners′ existing cognitive structures during the process of memorizing poems, thereby regulating the relevant cognitive load, reducing extraneous cognitive load, promoting the automation of schemas, and consequently facilitating the positive transfer of cognitive structures and ultimately enhancing memory efficiency and retention.

## Conclusion and outlook

This study employed a combined approach covering both controlled experiments and retrospective interviews to examine the effect of the schema-associated mnemonic method, based on the cognitive structure migration theory, on the efficiency and retention of classical Chinese poetry memorization. The results demonstrated that the experimental group outperformed the control group in both memory efficiency and retention, revealing that the method does work on promoting the positive transfer of learners′ cognitive structures, thereby enhancing the efficiency and retention of classical poem memorization. The findings of this study highlight the effective integration of classical Chinese poetry teaching with educational technology, providing support for educators and decision-makers. Generally speaking, this method can facilitate the connections of new materials with existing knowledge^20^ by means of association and imagination^14–16^, thereby reinforcing cognitive structures in turn^19^ and promoting active interaction between newly-acquired knowledge and pre-existing cognitive structures, ultimately helping to improve efficiency of memorization^18^. It can also evoke students′ emotions and enable them to memorize classical poems in an immersive manner, thus fostering emotional resonance with the poets^3,7,8^, which further promotes the automation of schemas, compensates for the limited capacity of working memory and lessens cognitive load, and eventually enhances memory efficiency^25^. Meanwhile, the "advance organizer" enables learners to enhance the availability and discriminability of their cognitive structures, thereby facilitating the positive transfer^17–20^ and reinforcing memory retention. Additionally, the mnemonic images in memory formation, through their association with existing cognitive schemata, promote cognitive assimilation^28^ and cognitive transfer^29^, thereby aiding in memory retention.

As required by the Standards^2^, Chinese courses should inspire and cultivate students′ passion for the Chinese language, and promote their balanced development through the subtle influence of outstanding cultures. Classical poetry, as a treasure of Chinese national culture, is undoubtedly full of the needed materials to help meet the above requirements. Therefore, in both teaching and learning activities, more attention should be paid to the accumulation and recitation of classical Chinese poems, emphasizing the role of "advance organizers", to assist students in connecting the to-be-memorized poems with their existing cognitive structures, and promoting the memory efficiency and retention. Therefore, in future teaching and learning activities, the schema-associated mnemonic method can be widely adopted, which is also a response to Ausubel′s statement that "*the most important single factor influencing learning is what the learner already knows*"^17^.

However, it is worth noting that this study also has its limitations, such as a small number of participants, a short memory time of 10 min, and the lack of a questionnaire survey to learn about the memory processes of all participants. Subsequent research can expand the number of participants, increase the memory time, and conduct further validation. Other than that, future research and relevant teaching activities should take into account the diversity of learners′ cognitive structures and push forward corresponding studies. Finally, further exploration is needed on how to maximize the alignment between mnemonic images and the original poetic fields of classical Chinese poems, and expand the application of the mnemonic method to other educational domains.

## Data Availability

The datasets generated during and/or analysed during the current study are available from the corresponding author on reasonable request.
